# The Current Status of Drug Repositioning and Vaccine Developments for the COVID-19 Pandemic

**DOI:** 10.3390/ijms21249775

**Published:** 2020-12-21

**Authors:** Jung-Hyun Won, Howard Lee

**Affiliations:** 1 Department of Molecular Medicine and Biopharmaceutical Sciences, Graduate School of Convergence Science and Technology, Seoul National University, Seoul 03080, Korea; junghyunwon97@gmail.com; 2 Center for Convergence Approaches in Drug Development, Graduate School of Convergence Science and Technology, Seoul National University, Seoul 03080, Korea; 3 Department of Clinical Pharmacology and Therapeutics, College of Medicine, Seoul National University, Seoul 03080, Korea; 4 Department of Clinical Pharmacology and Therapeutics, Seoul National University Hospital, Seoul 03080, Korea; 5 Department of Transdisciplinary Studies, Graduate School of Convergence Science and Technology, Seoul National University, Seoul 16229, Korea

**Keywords:** SARS-CoV-2, COVID-19, drug repositioning, vaccine, public health crisis

## Abstract

Since the outbreak of coronavirus disease 2019 (COVID-19) was first identified, the world has vehemently worked to develop treatments and vaccines against severe acute respiratory syndrome coronavirus 2 (SARS-CoV-2) at an unprecedented speed. Few of the repositioned drugs for COVID-19 have shown that they were efficacious and safe. In contrast, a couple of vaccines against SARS-CoV-2 will be ready for mass rollout early next year. Despite successful vaccine development for COVID-19, the world will face a whole new set of challenges including scale-up manufacturing, cold-chain logistics, long-term safety, and low vaccine acceptance. We highlighted the importance of knowledge sharing and collaboration to find innovative answers to these challenges and to prepare for newly emerging viruses.

## 1. Introduction

The outbreak of coronavirus disease 2019 (COVID-19) caused by the novel severe acute respiratory syndrome coronavirus 2 (SARS-CoV-2), which was first reported in Wuhan City, Hubei Province, China, on 31 December 2019, has spread over the world with an unparalleled speed [1,2]. The disease is so transmissible that the number of cases and deaths rose rapidly across borders, which prompted the World Health Organization (WHO) to declare the COVID-19 outbreak to be a global pandemic on 11 March 2020. As of 26 November 2020, more than 60,700,000 cases and 1,425,000 deaths have been reported worldwide [3].

Given the severity of the disease and the rapid rise in the number of affected individuals, therapeutics and vaccines to tackle COVID-19 are urgently needed. Scientists, governments, regulators, and pharmaceutical companies from every corner of the globe have ramped up together to develop drugs and vaccines against SARS-CoV-2 [4]. However, the therapeutic strategies to cope with SARS-CoV-2 infection have only played a supportive role until now [5]. Initially, the repositioning of existing drugs received much attention because it could be a short-cut compared with conventional, lengthy, and costly new drug development, but its effectiveness has not been satisfactory yet [6]. Therefore, prevention which aimed to reduce transmission while establishing a herd immunity using vaccines seems to be the key to ending the COVID-19 pandemic.

Vaccines against SARS-CoV-2 are being developed on an unprecedented fast track [4,7,8]. However, even if vaccines are successfully developed, there are still many questions with definitive answers that are unavailable. For example, how can we efficiently manufacture at least 16 billion doses of vaccine to cover the entire world assuming two doses are needed for each person, how will the vaccines be distributed to people in need with their quality being unaffected (i.e., when using cold-chain methods), and how are we going to raise public awareness of the vaccine and its necessity? Furthermore, vaccines may come too late to influence the first wave of the pandemic, although they might be useful for next waves and for a post-pandemic scenario. Certainly, humankind will learn important lessons from handling the global pandemic at this scale, which will allow us to be better prepared for the future public health crises.

The objectives of this review were three-fold. First, we briefly summarized up-to-date published clinical data of leading drug repositioning options and vaccine developments for COVID-19. Second, we discussed remaining challenges and issues we will face after a vaccine is successfully developed. Third, we stressed the importance of innovative, well-coordinated, long-term research to prepare for future public health crises. To better achieve those objectives, we first briefly described the characteristics of the coronavirus.

## 2. Coronavirus in Brief

The coronavirus is a positive single-stranded RNA virus (+ssRNA) with a crown-like appearance [9]. Viruses of the family Coronaviridae have been identified in, but not limited to, mammals, including mice, dogs, bats, and cats [10]. To date, several novel mammalian coronaviruses such as SARS (severe acute respiratory syndrome) and MERS (middle east respiratory syndrome) have been shown to be pathogenic to humans [10]. SARS-CoV-2 is a novel coronavirus belonging to the betaCoVs category [11]. SARS-CoV-2 is highly related to two bat-derived SARS viruses, bat-SL-CoVZC45 and bat-SL-CoVZXC21 with an 88% overlap of genome sequences [11]. SARS-CoV-2 also showed 79% and 50% overlap of genome sequences with SARS-CoV and with MERS-CoV, respectively [11]. Besides, the pangolin coronavirus, i.e., pangolin-CoV-2020, showed 90.32% overlap of its genome sequence with SARS-CoV-2 [12].

**Figure 1:**
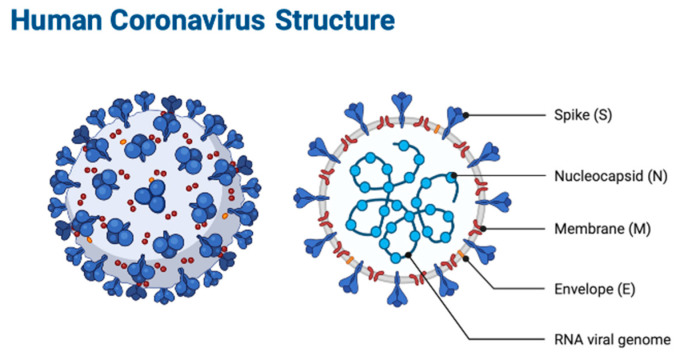
Structure of human coronavirus: the spike (S), envelope (E), membrane (M), and nucleocapsid (N) proteins. Created with BioRender (https://biorender.com/) [13].

The genome of SARS-CoV-2 encodes the spike (S), envelope (E), membrane (M), and nucleocapsid (N) proteins (Figure 1) [13]. The genomic sequence of SARS-CoV-2 encoding the S protein was highly related to the Bat-CoV-RaTG13 and pangolin-CoV-2020 with 93.15% and 84.52% overlap, respectively [12]. Each protein has its own functions, of which the spike and the envelope proteins have a pivotal role in virus pathogenicity. For example, the spike protein mediates viral invasion into host cells via the angiotensin-converting enzyme 2 (ACE2), and the envelope protein is involved in the formation and release of viral particles [13].

**Figure 2:**
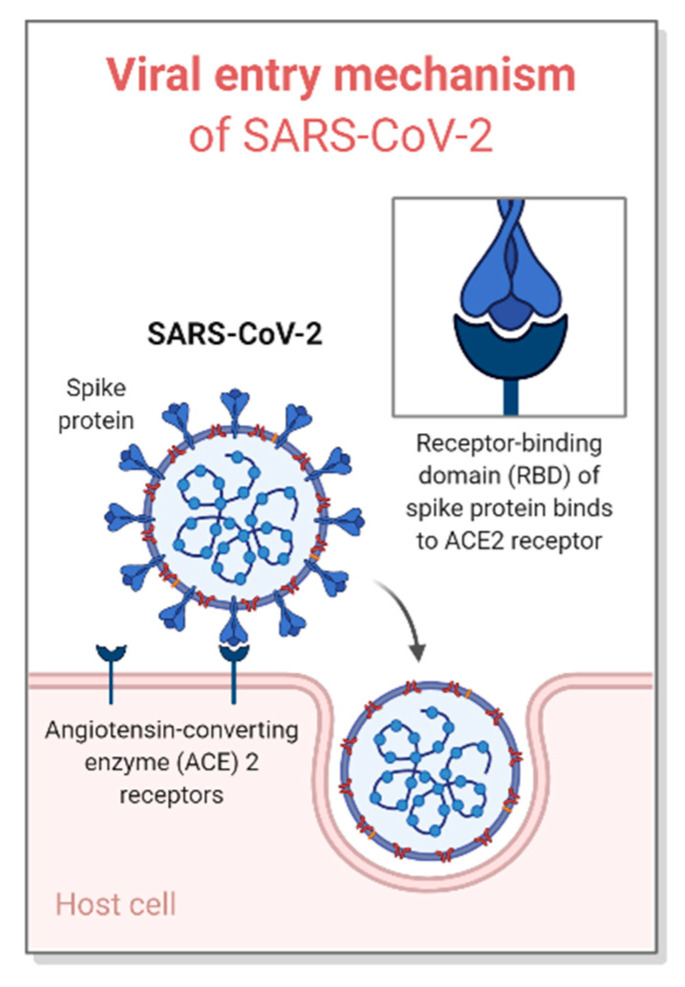
Viral entry mechanism of severe acute respiratory syndrome coronavirus 2 (SARS-CoV-2). SARS-CoV-2 first binds to ACE2 expressed in nasal epithelial cells or the respiratory tract and then penetrates into the target cell. Created with BioRender (https://biorender.com/) [16].

ACE2 is expressed in the bronchus, lung, heart, nasal mucosa, etc. [14]. When SARS-CoV-2 enters the respiratory tract, where ACE2 receptor binding sites are highly expressed, SARS-CoV-2 first binds to ACE2 and penetrates into the target cell [15]. Next, viral RNA is replicated by the RNA-dependent RNA polymerase (RdRp), and various viral proteins required to infect the host are produced by using the viral RNA [15]. A complete structure of SARS-CoV-2, composed of those replicated RNA and proteins, leaves the infected cell and eventually spreads throughout the body through successive proliferation (Figure 2) [15].

## 3. Drug Repositioning for SARS-CoV-2 Infection

At the time of writing this review, few treatments have been proven to be efficacious against SARS-CoV-2 infection. According to a living WHO guideline on drugs for COVID-19, many treatments against SARS-CoV-2 infection are only supportive and symptomatic, for example, ventilation, corticosteroids such as dexamethasone, and immunomodulatory drugs [5]. On the other hand, on 21 November 2020, the U.S. Food and Drug Administration (FDA) issued an Emergency Use Authorization (EUA) for REGN-CoV2, an antibody cocktail of casirivimab and imdevimab to adult patients with mild to moderate COVID-19 [17]. In the meantime, researchers are working enthusiastically to develop effective treatments for COVID-19. For this purpose, several antiviral drugs are being repositioned towards being used for COVID-19 treatments (Table 1). Among several antiviral drugs, WHO have supported remdesivir, lopinavir/ritonavir, and chloroquine/hydroxychloroquine for their repositioning potentials for COVID-19, which we describe below in detail [18].

### 3.1. Nucleoside Analogs

Nucleoside analogs such as favipiravir, ribavirin, and remdesivir are RdRp inhibitors for RNA viruses and induce premature termination of viral genome replication. Remdesivir is an adenosine analog, which shows antiviral activity by interfering the synthesis of new viral RNA with chain termination [19]. Although remdesivir was less effective than other antivirals in treating Ebola virus infection in a phase 3 trial [32], it has been actively studied for its potential to treat COVID-19. For example, the first COVID-19 patient treated with remdesivir in January 2020 had improved clinical conditions such as increased oxygen saturation value (94% to 96%) without any observed adverse events [33].

According to a phase 3 clinical trial enrolling 237 patients with severe COVID-19 (NCT04257656), randomly assigned to remdesivir or placebo in a 2:1 ratio, remdesivir was not associated with statistically significant clinical benefits (hazard ratio 1.23 [95% CI 0.87–1.75]) [34,35]. In contrast, according to another phase 3 clinical trial in 1059 adult patients hospitalized with COVID-19 (NCT04280705), remdesivir significantly lessened the median time to recovery compared to a placebo (11 vs. 15 days; rate ratio for recovery, 1.32 [95% CI 1.12–1.55]; *p* < 0.001) [36]. Additionally, remdesivir lowered mortality more than the placebo did, although the difference was not statistically significant (7.1% vs. 11.9% with remdesivir vs. placebo, respectively; hazard ratio for death, 0.70 [95% CI 0.47–1.04]) [36]. Various studies underway or in progress have recommended combined administration of remdesivir with other therapeutic options such as immunomodulatory drugs rather than remdesivir alone [36,37,38]. On 22 October 2020, the U.S. FDA approved remdesivir for the treatment of COVID-19 requiring hospitalization [39].

### 3.2. Protease Inhibitors

Viral proteases effectively cleave virus-encoded polyproteins, which is required for successful viral gene expression and replication [40,41]. Proteases inhibitors bind to the viral protease, thereby preventing virus replication [40,41]. In silico investigations using molecular docking-based screening of FDA-approved drugs have found that ritonavir, lopinavir, simeprevir, and asunaprevir may inhibit the main proteases of SARS-CoV-2 [22,42,43]. Lopinavir and ritonavir are protease inhibitors, and their combination was approved to treat patients with HIV infection [44,45,46]. Lopinavir is an inhibitor of HIV type 1 protease while ritonavir enhances lopinavir’s bioavailability by inhibiting its metabolic inactivation [23]. Previously, the combination of lopinavir and ritonavir showed antiviral activities against SARS and MERS [45,46], and therefore it was suggested and tested for the treatment of SAR-CoV-2 [47]. However, in a clinical trial (ChiCTR 2000029308), lopinavir-ritonavir combination failed to provide significant clinical improvements over standard care alone in 199 adult patients with severe COVID-19. (ratio for clinical improvement, 1.31 [95% CI 0.95–1.80]) [48]. Likewise, no benefit was observed in the reduction of viral RNA loads for severe SARS-CoV-2 patients (ratio, 1.01 [95% CI 0.76–1.34]) [48].

### 3.3. Chloroquine/Hydroxychloroquine

Chloroquine has been approved to treat malaria and autoimmune diseases such as lupus erythematosus and rheumatoid arthritis [49]. Chloroquine acts as an antiviral agent by interrupting endosomal acidification, and by inhibiting the glycosylation of ACE2, which are essential for virus-host cell fusion [22,27,28,50]. Previous studies demonstrated that chloroquine has broad antiviral activity against HIV, Ebola, SARS, MERS, and Nipah viruses [28,50,51,52]. Besides, chloroquine showed effective clinical outcomes in the treatment of COVID-19 associated pneumonia [27,53]. Based on these findings, the U.S. FDA granted an EUA for the use of chloroquine and hydroxychloroquine against SARS-CoV-2 infection [54]. However, a few studies have raised concerns about the FDA’s decision [55]. For example, in a phase 3 clinical trial of hydroxychloroquine as a post-exposure prophylactic measure against COVID-19 (NCT04308668), hydroxychloroquine did not prevent the disease progression of participants who had close contact with someone diagnosed with COVID-19 [56]. The number of participants who experienced any new symptoms related to COVID-19 after administration of hydroxychloroquine or a placebo did not differ between the hydroxychloroquine and placebo groups (11.8% vs. 14.3% with hydroxychloroquine and placebo, respectively; absolute difference, −2.4 percentage points; [95% CI −7.0–2.2]; *p* = 0.35) [56]. Although no serious adverse reaction was reported in this study, adverse events such as nausea, loose stools, and abdominal discomfort were observed significantly more frequently in the hydroxychloroquine group (40.1% vs. 16.8% in hydroxychloroquine and placebo, respectively; *p* < 0.001) [56]. Furthermore, in a phase 2b clinical trial enrolling 81 patients with severe COVID-19 (NCT04323527), randomly assigned to high- or low-dose of chloroquine in a 1:1 ratio, the high-dose group experienced the prolongation of the QTc interval (>500 ms) more frequently than the low-dose group (18.9% vs. 11.1% with high- and low-dose groups, respectively). Lethality was 39.0% and 15% in the high- and low-dose groups, respectively [57]. Based on these results, the FDA provided warnings regarding the use of chloroquine or hydroxychloroquine in COVID-19 patients [58].

To sum up, the scorecard of drug repositioning for COVID-19 does not look promising so far, although it has received much attention initially. Most of the repositioned drugs failed to show significantly better efficacy than the control or placebo in treating COVID-19 patients, particularly when administered alone. The safety of the repositioned drugs for COVID-19 has not been reassuring either, particularly for chloroquine and hydroxychloroquine.

## 4. Vaccines for SARS-CoV-2 Prevention

Safe and effective vaccines against SARS-CoV-2 will be a key to overcoming the global pandemic, particularly when few treatments including the repositioned drugs have been shown to be safe and effective for COVID-19. New vaccine development typically takes >15 years, but SARS-CoV-2 vaccines are being tested on an unprecedented fast track [7]. Many have forecasted that SARS-CoV-2 vaccines could be developed in as short as 15–18 months [4,7]. To support their predictions, as of December 2020, more than 140 vaccine candidates are under development with 15 of them tested in phase 3 [59]. Those vaccine candidates can be grouped into five different platforms: protein subunit vaccines, viral-vectored vaccines, nucleic acid vaccines (mRNA and DNA vaccines), inactivated viruses, and live attenuated viruses. Each platform has its strengths and limitations (Table 2).

### 4.1. Protein Subunit Vaccines

Protein subunit vaccines contain recombinant antigenic proteins from COVID-19 such as the spike protein [60,61]. Protein subunit vaccines are safe and have fewer side effects than other vaccine platforms [61]. Furthermore, protein subunit vaccines are scalable for mass production under good manufacturing practice (GMP) standards [62]. However, protein subunit vaccines often require booster administrations, adjuvant, or a higher dose to enhance immune responses [63].

Protein subunit vaccines account for 50% of the vaccines currently under development for COVID-19 [64]. NVX CoV-2373 (Novavax) is a recombinant spike protein with the Matrix-M™ adjuvant [65,66]. In May 2020, Novavax launched a phase 1/2 randomized, observer-blinded, placebo-controlled clinical trial in healthy adults (NCT04368988), where NVX CoV-2373 elicited an antibody response four-fold greater than the mean titer in human COVID-19 convalescent serum [67]. Mild headache, fatigue, and myalgia were the most common systemic events [67]. Mild reactogenicity was also noted, but no medical intervention was required and no drop out due to reactogenicity was reported [67]. A phase 2 trial with NVX CoV-2373 is on-going in South Africa, which enrolled 2904 volunteers (NCT04533399) [68]. Furthermore, a phase 3 trial with NVX CoV-2373 is underway in the United Kingdom with a target number of 9000 (NCT04583995) [69]. The clinical trials with NVX CoV-2373 have been funded by the Coalition for Epidemic Preparedness Innovations (CEPI) [70]. In July 2020, Novavax was chosen to participate in the Operation Warp Speed, a U.S government sponsored program [71].

The University of Queensland was developing a protein subunit vaccine with a molecular clamp technology [72,73]. The molecular clamp is a surface protein that improves the recognition of the correct antigen, which can enhance immune responses [73]. Previously, the University of Queensland entered a partnership with CEPI and started a phase 1 clinical trial in July 2020 [72]. However, on 10 December 2020, the Australian government put a hold on the University of Queensland vaccine, which produced Human Immunodeficiency Virus-false positives [74].

Clover Biopharmaceuticals is developing SCB-2019, a COVID-19 S-Trimer subunit vaccine with adjuvant systems of GSK and Dynavax. Clover Biopharmaceuticals launched a phase 1 clinical trial (NCT04405908) in June 2020, and is planning for a global phase 2b/3 trial [8,75].

### 4.2. Viral-Vectored Vaccines

Viral-vectored vaccines are genetically engineered viruses that deliver viral genetic materials into cells [76,77]. Viral-vectored vaccines could enhance a wide range of immune responses without adjuvants [76,78]. However, the host, i.e., vaccine recipients, may have pre-existing immunity against the vector through prior exposure to the virus, eventually reducing the vaccine’s efficacy or immunogenicity [77]. Measles virus, adenovirus vectors, vesicular stomatitis virus, and modified vaccinia Ankara are the most commonly used viral vectors [79].

Janssen Pharmaceutical Companies of Johnson & Johnson is developing Ad26.CoV2-S, an adenovirus-vectored vaccine. Johnson & Johnson can provide a large-scale production of vaccines through the AdVac^®^ and PER.C6^®^ technologies, which are cost-effective manufacturing systems that were used for the Ebola vaccine [80,81]. In the second half of July 2020, Johnson & Johnson initiated a phase 1/2a clinical trial with Ad26.CoV2-S in the U.S. and Belgium (NCT04436276) [82]. At the time of writing this review, phase 1 (NCT04509947), phase 2a (NCT04535453), and phase 3 (NCT04505722) clinical trials are on-going simultaneously [82]. On 12 October 2020, Johnson & Johnson temporarily stopped the phase 3 clinical trial, which had a target number of 60,000 healthy adults, due to an unexplained adverse event in one participant [83]. After a thorough evaluation of the adverse event, however, Johnson & Johnson resumed the trial [83].

AstraZeneca and the University of Oxford are co-developing AZD1222 (ChAdOx1 nCoV-19), a chimpanzee adenovirus-vectored vaccine that expresses the spike protein of SARS-CoV-2 [84]. The University of Oxford could have accelerated the development of AZD1222 because of their past research experience in developing ChAdOx1 MERS, a vaccine candidate against MERS-CoV [85]. A single administration of AZD1222 induced a robust humoral and cellular immune responses in rhesus macaques. Virus-specific neutralizing antibodies were detected in all vaccinated animals (VN titer, 5–40), while none were detected in control animals [86]. On 23 April 2020, AstraZeneca launched a randomized, single-blind, phase 1/2 trial (NCT04324606) that enrolled 1090 healthy adults in UK. After the first vaccination with AZD1222, the concentrations of antibodies specific to SARS-CoV-2 spike protein reached peak by day 28 (median, 157 ELISA units (EU); interquartile range, 96–317; *n* = 127) [87]. In participants who received a booster dose, the concentrations of antibodies specific to SARS-CoV-2 spike protein elevated to a median of 639 EU (360–792, *n* = 10) at day 56 [87]. Mild to moderate fatigue and headache were the most common systemic events reported in the AZD1222 group, and one serious adverse event, hemolytic anemia, occurred in the control group (who received a meningococcal conjugate vaccine) [87]. A phase 2/3 trial (NCT04400838, target number = 12,390) and a phase 3 trial (NCT04516746, target number = 40,051) are on-going to support large-scale evaluation of AZD1222 [88]. On 6 September 2020, AstraZeneca temporarily paused the phase 3 study after a suspected case of transverse myelitis was seen during the trial [89]. In September 2020, the phase 3 clinical trials with AZD1222 resumed enrollment in the UK, Brazil, South Africa, India, Japan, and eventually in the U.S. on 23 October 2020 [90]. The non-peer-reviewed results showed that AZD1222 was more efficacious than placebo in preventing COVID-19 by 90% (*n* = 2741, low-dose followed by high-dose, *p* ≤ 0.0001) and 62% (*n* = 8895, two high-doses, *p* ≤ 0.0001) [91]. No serious adverse events were observed in participants treated with AZD1222 [91].

Gamaleya Research Institute in Russia has been developing Sputnik V (formerly Gam-Covid-Vac), an adenovirus-vectored vaccine that carries the gene for the spike protein of SARS-CoV-2 [92]. The clinical trials with Sputnik V have been funded by the Russia Direct Investment Fund [92,93]. In June 2020, Gamaleya launched a phase ½ prospective, non-randomized clinical trial in healthy adults (NCT04436471 and NCT04437875) [94,95]. SARS-CoV-2 RBD-specific IgGs were detected in all participants after 21 days after vaccination of Sputnik V [92]. Mild to moderate pain at injection site and hyperthermia were the most common adverse event of Sputnik V [92]. The phase 3 trials of Sputnik V are on-going in Russia (NCT04530396, target number = 40,000) [96], Belarus (NCT04564716, target number = 100) [97], and Venezuela (NCT04642339, target number = 2000) [98]. The non-peer-reviewed results showed that Sputnik V is 91.4% effective in preventing COVID-19 without any serious adverse events [99]. The analysis was based on 39 confirmed cases of COVID-19 (31 vs. 8 cases in the placebo and vaccine groups, respectively) [99]. Besides, on 11 December 2020, AstraZeneca which is also developing an adenovirus-vectored vaccine, accepted the Russia Direct Investment Fund’s proposal to test the safety and immunogenicity of combination of AZD1222 and Sputnik V [100,101].

### 4.3. Nucleic Acid Vaccines (mRNA and DNA)

Nucleic acid vaccines refer to either mRNA or DNA vaccine [102,103], accounting for 37% of the vaccines under development for COVID-19. Nucleic acid vaccines are cost-effective and easy to manufacture [64,104]. Nucleic acid vaccines directly inject fragments of viral mRNA or plasmids that encode antigens necessary for immune responses; therefore, there is no risk of infection or direct virulence by vaccines [104].

Pfizer and BioNTech are jointly developing two codon-optimized mRNA vaccine candidates: BNT162b1 encoding the receptor-binding domain (RBD), and BNT162b2 as the spike protein of SARS-CoV-2 [105]. A phase 1/2/3, placebo-controlled, observer-blind, dose finding, vaccine candidate selection, and efficacy study of BNT162b1 and BNT162b2 (NCT04368728) is on-going [105]. BNT162b1 (10-μg dose level) raised RBD-specific IgG concentrations by 35 days after the second dose, results similar to those observed in human convalescent serum (5880-16,166 U ml^−1^ vs. 602 U ml^−1^ in the BNT162b1 group and human convalescent serum, respectively) [106]. Mild to moderate fatigue and headache were the most common systemic events and no serious adverse event was reported in the BNT162b or placebo group [106]. Antibody titers were comparable between BNT162b1 and BNT162b2, but BNT162b2 was selected for further studies partly because it showed a more favorable safety profile than BNT162b1, particularly in older subjects [107]. As of 18 November 2020, a phase 3 trial of BNT162b2 has enrolled 43,661 participants and showed 95% efficacy in the vaccination group in preventing COVID-19 (*p* < 0.0001) [108]. The analysis was based on 170 confirmed cases of COVID-19 (162 vs. 8 cases in the placebo and vaccine groups, respectively) [108]. No serious adverse events were observed in participants treated with BNT162b2 [108]. On 20 November 2020, Pfizer and Biotech submitted an EUA to the U.S. FDA [109]. On 11 December 2020, FDA issued an EUA of BNT162b2 for the prevention of COVID-19 [110]. Besides, BNT162b2 received regulatory approvals in Canada, the UK, Bahrain, and Saudi Arabia [59,111,112].

Moderna, in a partnership with the National Institute of Allergy and Infectious Diseases (NIAID), is developing mRNA-1273, which expresses the spike protein of SARS-CoV-2 [4,113]. In March 2020, Moderna initiated a phase 1, open-label, dose-ranging study of mRNA-1273 in 120 healthy adults (NCT04283461). In this study, the antibody response provided by mRNA-1273 was increased as the vaccine dose was increased, with antibody titers being higher than that in the convalescent serum specimens (anti–S-2P antibody geometric mean titer (GMT) on day 57, 299,751 vs. 782,719 vs. 1,192,154 vs. 142,140 in the 25-μg, 100-μg, 250-μg, convalescent serum groups, respectively) [114]. With a Fast Track designation by the U.S. FDA and financial support from CEPI for the development of mRNA-1273, Moderna initiated a phase 2 (*n* = 600, NCT04405076) and a phase 3 clinical trial (*n =* 30,000, NCT04470427) [113]. The phase 3 trial of mRNA-1273 showed 94.1% vaccine efficacy in preventing COVID-19 without any significant safety concern [115]. The analysis was based on 196 confirmed cases of COVID-19 (185 vs. 11 cases in the placebo and the mRNA-1273 groups, respectively) [115]. On 30 November 2020, Moderna submitted an EUA to the U.S. FDA [115]. Furthermore, on 2 December 2020, Moderna launched a phase 2/3 trial (NCT04649151) to test mRNA-1273 in adolescents between 12 and 18 years of age [116].

Inovio Pharmaceuticals is developing INO-4800, a prophylactic DNA vaccine that encodes the spike protein of SARS-CoV-2 [117]. Inovio Pharmaceuticals started a phase 1 trial (NCT04336410) in April 2020, and a phase 1/2a trial (NCT04447781) in July 2020 [117].

### 4.4. Others: Live Attenuated or Inactivated Vaccines

Live attenuated or inactivated vaccines are an old, but effective platform. They are manufactured by weakening or killing pathogens, respectively, so that the vaccines become harmless before entering the host [118]. As the most classic platform, live attenuated or inactivated vaccines account for the majority of approved human vaccines [119]. For example, vaccines for hepatitis A, rabies, whole-cell pertussis are based on inactivated vaccines [119]. However, live attenuated or inactivated vaccines require the use of adjuvants, and is expensive to manufacture [120]. Moreover, live attenuated vaccines may revert to the virulent organism. Live attenuated or inactivated vaccines account for 17% of the vaccines under development for COVID-19 [64].

Sinovac Biotech is developing CoronaVac, a vaccine containing inactivated SARS-CoV-2 [121]. In April 2020, Sinovac Biotech launched a phase ½ clinical trial with CoronaVac (NCT04352608, *n* = 743) [121,122]. In participants who received a second dose, the seroconversion rates of neutralizing antibodies to live SARS-CoV-2 were 97% (GMT 44.1 [95% CI 37.2–52.2]) and 100% (GMT 65.4 [95% CI 56.4–75.9]) in the 3 μg and 6 μg groups, respectively [121]. Most of the adverse events of CoronaVac were mild, and the most common symptom was injection-site pain [121]. In July 2020, the Chinese government granted an emergency approval for limited use of CoronaVac [121]. The phase 3 trials of CoronaVac are on-going in Indonesia (NCT04508075, target number = 1620) [123], Brazil (NCT04456595, target number = 13,060) [124], and Turkey (NCT04582344, target number = 13,000) [125].

## 5. Remaining Challenges

Pfizer’s BNT162b2 has received regulatory approval in a few countries [110,111,112], and several vaccine candidates are likely to be ready soon for regulatory approval and mass rollout [59]. However, this will lead to a whole new set of challenges for effective vaccination: scale-up manufacturing, cold-chain logistics, long-term safety, and poor vaccine acceptance (i.e., low public awareness of the vaccine and its necessity). Assuming that 60% of antibody-positive people are required to achieve herd immunity, >16 billion doses of vaccines are needed globally. However, the paucity of appropriate and qualified production facilities worldwide makes the scale-up production of COVID-19 vaccines challenging. Researchers from the Center for Global Development have modeled manufacturing processes to estimate the time needed to manufacture COVID-19 vaccines and supply them to meet the global demand [126]. Through the modeling, the world can plan a detailed timeline for the successful vaccine strategy [126].

What makes the situation worse is that most of the promising vaccine candidates are using the latest platforms such as nucleic acid or viral vectors that have never been mass-produced before [61,102,103]. Establishing cold-chain logistics and storage is another challenge for vaccines against COVID-19 [127,128]. Like other biologics, vaccines are temperature-sensitive; their stability and safety will be compromised if exposed to unwanted temperature [127,128]. Leading vaccine candidates, particularly mRNA vaccines, must be stored and transported at a very low temperature. For example, Pfizer’s BNT16 requires long-term storage at −70 °C ± 10 °C and Moderna’s mRNA-1273 must be shipped at −20 °C [129,130]. To address those manufacturing and cold-chain issues, Gavi, CEPI, and WHO formed a coalition to accelerate the development and manufacture of COVID-19 vaccines and to supply them around the world [131].

Not only the efficacy, but also the safety of the vaccine has to be shown through large, long-term clinical trials for regulatory approval [132]. However, the unprecedentedly accelerated development of COVID-19 vaccines has raised concerns that they could be approved with incomplete data, particularly for long-term safety in various populations [131]. For example, China approved CanSino Biologic’s COVID-19 vaccine candidate before the phase 3 trials had been finished [59,133,134]. Since then, the vaccine has been administered to soldiers in the military [59,133,134]. Although no serious adverse events have been reported among COVID-19 vaccine candidates, the clinical data of the vaccine should be reviewed in-depth continuously to ensure the long-term safety and efficacy of the vaccine. In addition, the public should be fully informed before vaccination about long term adverse events of the vaccine.

Public awareness and acceptance of the vaccine is another challenge. According to a public survey conducted in 19 countries (*n =* 13,426), only 31.9% of the respondents said they would get shots of a COVID-19 vaccine [135]. Many people are still skeptical about the utility of the vaccine, and therefore reluctant to vaccination. This may be partly attributed to the misinformation or lack of scientifically justifiable information publicly available. To improve the trust in COVID-19 vaccines and to increase their acceptance by the public, healthcare professionals, policymakers, and stakeholders should strive for developing messages for the public that encourage vaccination, while mitigating the harm of scientifically unjustifiable propaganda that asserts the futility of COVID-19 vaccines. At the same time, the public should assume their own responsibility, as global citizens, for establishing a safety net against COVID-19 by actively participating in community wide vaccination programs.

## 6. Conclusion

In each and every decade of the 21st century, we have undergone coronavirus pandemics: *SARS* in the 2000s, MERS in the 2010s, and COVID-19 in 2019 and onwards. Certainly, COVID-19 will not be the last pandemic in this century; newly emerging viruses, unprecedented and unheard of before, will continue threatening our lives. When facing these threats, governments, academia, and researchers have gathered together to develop treatments and vaccines against SARS-CoV-2, which is unparalleled too. More knowledge sharing, collaboration, and collective know-how from every corner of the globe will bring an innovative answer to the question that we are facing: if, when, and how we end COVID-19?

## Figures and Tables

**Table 1:** List of potential antiviral drugs that may be repositioned towards coronavirus disease 2019 (COVID-19) treatments.

Candidate	Target	Mechanisms	References
Remdesivir	RdRp	Interferes with virus RNA polymerases to inhibit virus replication.	[19]
Ribavirin	RdRp	Inhibits nucleotide biosynthesis by inhibiting viral RNA polymerase.	[20]
Favipiravir	RdRp	Inhibits viral transcription and replication.	[21,22]
Lopinavir/Ritonavir	Viral main proteases	Inhibits the viral proteases and blocks the multiplication.	[23,24]
Darunavir	Viral main proteases	Inhibits virus maturation by inhibiting the viral protease.	[25,26]
Chloroquine/Hydroxychloroquine	Viral entry	Changes endosomal pH; Inhibits viral entry and interferes with RdRp function.	[27,28,29]
Arbidol	Spike glycoprotein	Inhibits viral entry and post-entry stages.	[30,31]

RdRp, RNA-dependent RNA polymerase.

**Table 2:** Overview of Selected COVID-19 Vaccine Candidates.

Platform	Developer	Vaccine Candidate	Status *
Protein subunit vaccine	Novavax	NVX-CoV-2373	Phase 3
	The University of Queensland	SARS-CoV-2 Sclamp	Abandoned
	Clover Biopharmaceuticals	SCB-2019	Phase 1
Viral-vectored vaccine	Johnson & Johnson	Ad26.CoV2-S	Phase 3
	AstraZeneca, University of Oxford	AZD1222	Phase 2, Phase 3, Combined phases
	Gamaleya Research Institute	Sputnik V	Phase 3, Early use in Russia
mRNA vaccine	Pfizer, BioNTech	BNT162b2	Phase 2, Phase 3, Approved in Canada and other countries, Emergency use in U.S.
	Moderna, NIAID	mRNA-1273	Phase 3, Under FDA review.
DNA vaccine	INOVIO Pharmaceuticals	INO-4800	Phase 2
	Genexine	GX-19	Phase 1
Inactivated Vaccine	Sinovac Biotech	CoronaVac	Phase 3, Limited use in China.
	The Chumakov Center at the Russian Academy of Sciences	Whole-virion vaccine	Phase 1, Phase 2, Combined phases

* Updated 13 December 2020. NIAID, National Institute of Allergy and Infectious Diseases.

## Abbreviations

COVID-19	Coronavirus disease 2019
SARS-CoV-2	Severe acute respiratory syndrome coronavirus 2
WHO	World health organization
+ssRNA	Positive single-strand RNA virus
MERS	Middle east respiratory syndrome
ACE2	Angiotensin-converting enzyme 2
RdRp	RNA-dependent RNA polymerase
FDA	Food and Drug Administration
EUA	Emergency use authorization
GMP	Good manufacturing practice
CEPI	Coalition for epidemic Preparedness Innovations
EU	ELISA units
NIAID	National institute of allergy and infectious diseases
GMT	Geometric mean titers
